# Supplementary material for: Transcriptome Profiling of the Dorsomedial Prefrontal Cortex in Suicide Victims
Source: Int J Mol Sci. 2022 Jun 25;23(13):7067. doi: 10.3390/ijms23137067 (PMC9266666; doi:10.3390/ijms23137067)
Supplement: Supplementary file 1 [file ijms-23-07067-s001.zip › supplementary_material_legend-D¿«ra-mod.pdf]

SUPPLEMENTARY MATERIAL

Figure legends

Supplementary Figure S1. Characteristics of samples.

Mean age, gender and PMI proportions of samples in control (n=8) and suicide (n=8) groups. There were no significant differences between the groups for any covariates tested by Welch's unequal variances t-test (age, PMI) and Chi-square test (gender). (Age: $p=0.1$; Gender: $p = 0.59$; PMI: $p = 0.25$)

Supplementary Figure S2. Validation of the RNA-seq results with quantitative PCR.

The expression level changes in suicide victims are shown as determined by RT-qPCR. The mRNA expression of upregulated genes is elevated (A and B), while the expression of downregulated genes is reduced (C-E) in suicide victims, which confirms the expressional changes in gene expression profiles deduced from RNA-seq results. Bar graphs represent mean ± SEM of 8 control and 8 suicide individuals (*$p < 0.05$, **$p < 0.01$).

Supplementary Figure S3. Preparation and visualization of NECAB2 probe in the DMPFC.

A two-step polymerase chain reaction (PCR) was performed and PCR products were examined by agarose gel electrophoresis. The products of NECAB2 from the first PCR (1) and products of NECAB2 containing the T7 promoter from the second PCR (2) are indicated. The exact molecular weight of the NECAB2 product is 360 bp.

Supplementary Tables:

Supplementary Table S1. Gene mapping, read data, quality of sequencing.

Supplementary Table S2. Primer sequences of validated genes.
