# Supplementary figures and images for: Transcriptome Profiling of the Dorsomedial Prefrontal Cortex in Suicide Victims
Source: Int J Mol Sci. 2022 Jun 25;23(13):7067. doi: 10.3390/ijms23137067 (PMC9266666; doi:10.3390/ijms23137067)

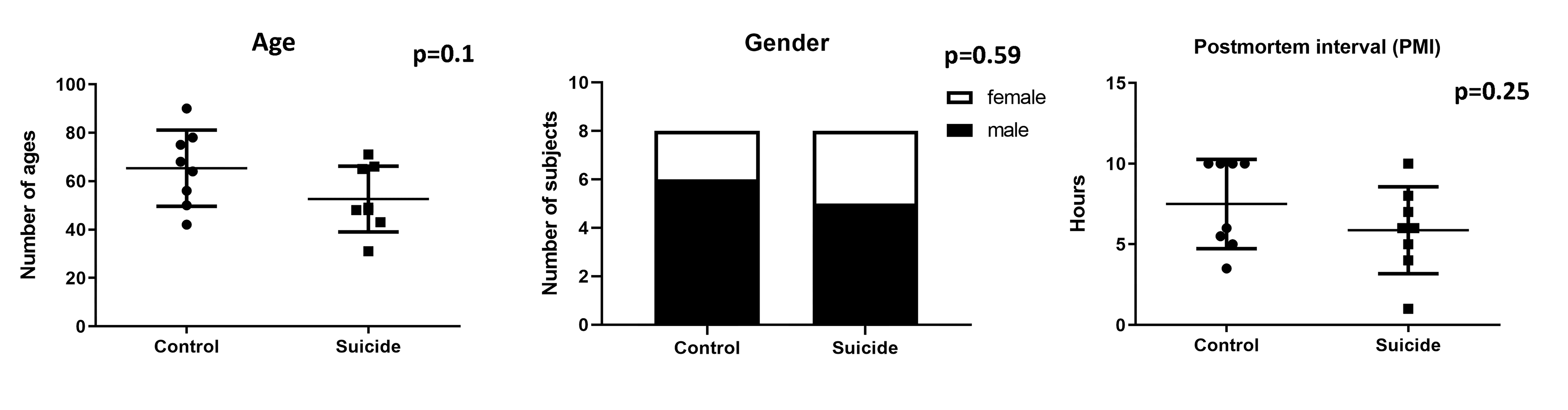

Supplement: Supplementary file 1 [file ijms-23-07067-s001.zip › S.Fig_S1.tif]

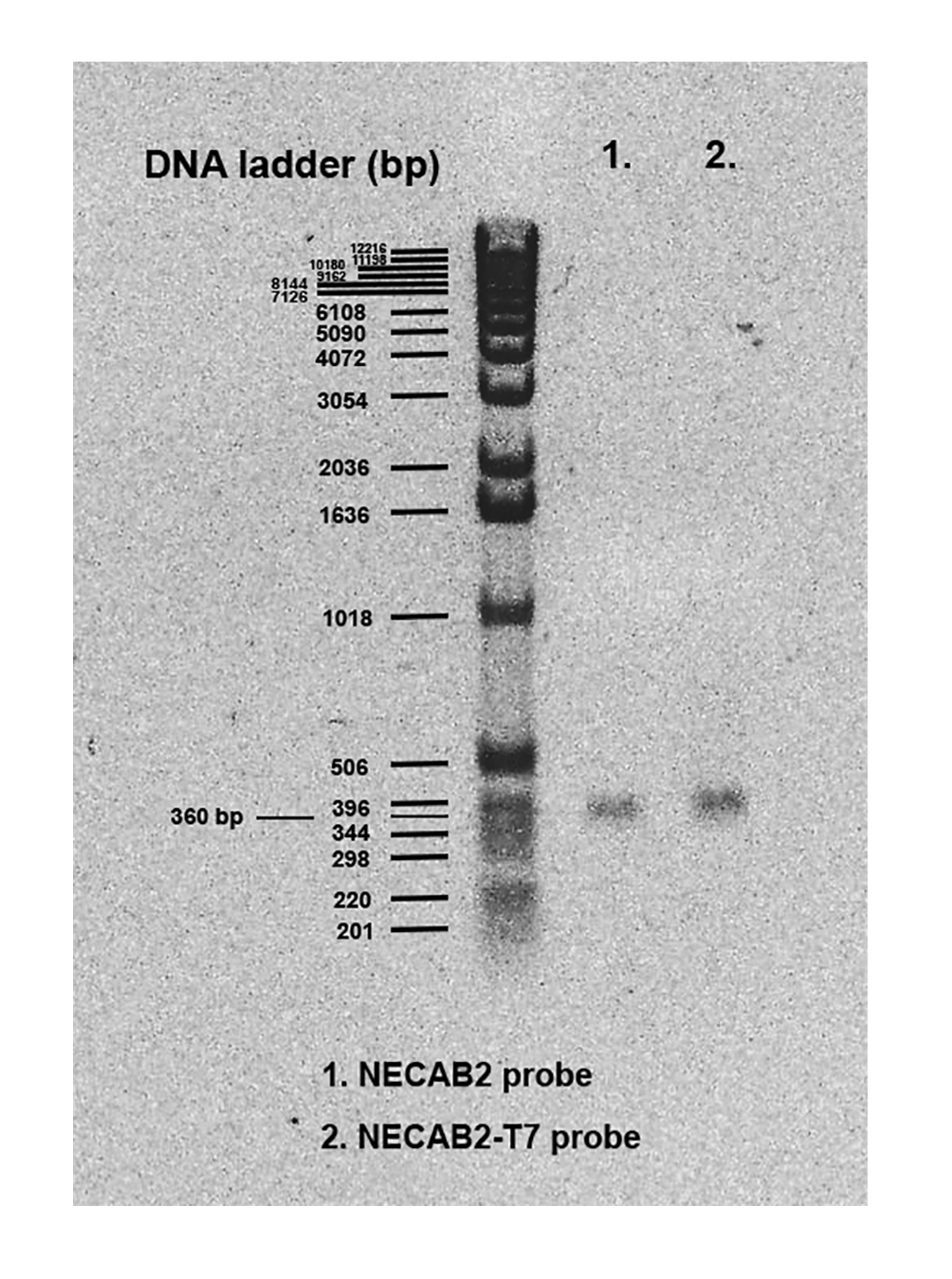

Supplement: Supplementary file 1 [file ijms-23-07067-s001.zip › S.Fig_S3.tif]
